# Iodide of Calomel

**Published:** 1857-09

**Authors:** 


*Iodide of Calomel.*—In the British and Foreign Medico-Chirurgical Review, we find the Iodide of Calomel recommended as an effectual remedy for engorged and ulcerated conditions of the neck of the uterus. It is applied by means of a pledget in the form of a pommade. "In the days succeeding the dressing, the exuded coagulum is detached by degrees, the volume of the os uteri diminishes, and becomes less than it was before the topical application; if there was any induration, which is generally the case, this induration is much less from the day succeeding the dressing. At the end of eight, ten, or twelve days, if this amelioration has made no progress, the dressing is renewed, and gives rise to the same phenomena, although in a less marked degree; and after two, three, four or five applications made at the same intervals, the os is usually restored to its normal volume, and the ulcerations are cicatrized."—*Recommended by Dr. Rochard in the L' Union Medicale, January 6th,* 1857.

